# The effect of a medical opinion on self-perceptions of weight for Mexican adults: perception of change and cognitive biases

**DOI:** 10.1186/s40608-017-0152-6

**Published:** 2017-05-03

**Authors:** Jonathan F. Easton, Christopher R. Stephens, Heriberto Román Sicilia

**Affiliations:** 1 C3–Centro de Ciencias de la Complejidad, Universidad Nacional Autónoma de México, Circuito Centro Cultural s/n, Ciudad Universitaria, Col. Universidad Nacional Autónoma de México, Del. Coyoacán, C.P. 04510 Ciudad de México, Mexico; 2 Instituto de Ciencias Nucleares, Universidad Nacional Autónoma de México, Circuito Exterior s/n, Ciudad Universitaria, Col. Universidad Nacional Autónoma de México, Del. Coyoacán, A. P. 70-543, C.P. 04510 Ciudad de México, Mexico

**Keywords:** BMI, Obesity, Linear regression, Cognitive biases, Self-perception, Medical Identification, Public Health Survey

## Abstract

**Background:**

This study analysed the relationship between perceived and actual Body Mass Index (BMI) and the effect of a prior identification of obesity by a medical professional for adults using difference in response for two distinct BMI self-perception questions. Typically, self-perception studies only investigate the relation with current weight, whereas here the focus is on the self-perception of weight differences.

**Methods:**

A statistical approach was used to assess responses to the Mexican ENSANUT 2006 survey. Adults in the range of BMI from 13 to 60 were tested on responses to a categorical question and a figure rating scale self-perception question. Differences in response by gender and identification of obesity by a medical professional were analysed using linear regression.

**Results:**

Results indicated that regardless of current BMI and gender, a verbal intervention by a medical professional will increase perceived BMI independently of actual BMI but does not necessarily make the identified obese more accurate in their BMI estimates. A shift in the average self-perception was seen with a higher response for the identified obese. A linear increase in perceived BMI as a function of actual BMI was observed in the range BMI < 35 but with a rate of increase much less than expected if weight differences were perceived accurately.

**Conclusions:**

Obese and overweight Mexican adults not only underestimated their weight, but also, could not accurately judge changes in weight. For example, an increase of 5 kg is imagined, in terms of self-image, to be considerably less. It was seen that an identification of obesity by a health care professional did not improve ability to judge weight but, rather, served as a new anchor from which the identified obese judge their weight, suggesting that even those identified obese who have lost weight, perceive their weight to be greater than it actually is. We believe that these results can be explained in terms of two cognitive biases; the self-serving bias and the anchoring bias.

## Background

A report from the Food and Agriculture Organization of the United Nations published in 2013 showed that for the first time Mexico has overtaken the USA as the country with the highest obesity rate in the World [1]. At 32.8%, one third of the Mexican population is obese, a figure that increases to approximately 70% including the overweight [2]. Obesity is a growing problem, affecting all parts of the world [3]. Consequently, obesity has become an important risk factor for the most common causes of death and illness worldwide, surpassing malnutrition and infectious diseases, as highlighted by Kopelman [4]. As obesity is such an important health risk, there are a large number of potential treatments and interventions available, as discussed in various summary articles, for example [5, 6]. However, a key problem, as will be seen here and as has been shown elsewhere [7], is that obesity is a condition frequently not recognised by the obese themselves, thus potentially delaying action, either by the individual or through the advice of a healthcare professional. For instance, although the idea of eating less and exercising more is recognised as beneficial, based on previous evidence [8, 9] it is apparent that without a consultation with a medical professional, there is a substantially lower probability that an adult will apply, or feel the need to apply, the changes necessary in order to avoid weight gain or lose weight.

A major factor linked to lack of self-awareness about obesity is the systematic bias associated with the self-perception of body weight. The discrepancy between actual and perceived BMI has been extensively reported, with studies from Europe [10], the United States [11–14] and Asia [15]. The main conclusion is that there is a systematic tendency for the overweight and obese to underestimate their actual BMI. Many studies consider how different factors affect the self-perception of BMI, such as level of education and type of student [10], amongst many other factors [16]. Several studies also exist for the Mexican population with differing points of emphasis. Adult-based investigations are mainly divided into separate male and female studies that then include other conditions, including socioeconomic status [17] and age [18, 19]. Several studies have compared similar groups between Mexico and the United States [20–22]. In these cases, identification of obesity was examined specifically for either obese males or females and for responses to one specific type of self-perception question.

Accepting obesity as a serious condition, it is imperative that people can identify it both in themselves and in others. The potential public health consequences of the misperception of BMI and the inability to recognise obesity in oneself have also been the subject of much discussion [23–27]. In particular, Duncan et al. [23] have shown that the misperception of BMI among overweight and obese adults was associated with less likelihood of interest in, or attempts at, weight loss, and less physical activity. As they point out, their findings are consistent with the Health Belief Model [28], which proposes that a perceived susceptibility to a given condition is necessary to promote appropriate behavioural changes. Thus, correcting perception of weight status may be an important consideration in the design of weight loss interventions. It has also been shown that an identification of obesity by a healthcare professional [17, 29] is correlated to a lower degree of misperception of BMI among the overweight and obese. What is not clear, however, is if this leads to better outcomes in terms of weight reduction and/or healthier lifestyles, though there is some evidence that awareness of obesity can have a positive effect [30].

In this paper, we considered the relationship between actual and self-perceived BMI as a function of gender and of a previous identification of obesity by a healthcare professional for a large, representative Mexican adult population, over all BMI and age ranges and for two different forms of self-perception question. The data used in this analysis is associated with the Mexican National Health and Nutrition Survey (ENSANUT) 2006 [31]. This is a nationwide survey containing data from all states in Mexico covering a wide range of topics including social demographics, home life, nutrition, work, education, medical histories and much more.

The principal aim of this paper was to compare the relationships between actual and perceived BMI, as well as BMI differences, using two different self-perception questions: a categorical BMI status question and a self-perception figure rating scale (FRS) question. Further aims were to determine the impact of a consultation identifying obesity with a healthcare professional on perceived BMI, and, finally, to examine the effect of gender.

Based on the studies discussed above [14–20, 27], we hypothesised that body weight is underestimated for a complete population for multiple perception questions and that an intervention from a medical professional can cause a substantial change in self-perception.

## Methods

From the ENSANUT 2006 survey, we took 20360 adults (age 20 or above) with nutritional data as the full data set. Exclusions included any adult with missing data, or responses of ‘unknown’ and ‘don’t know’ in any of the required variables, such as: height or weight, medically identified obesity and the weight self-perception questions. Adults who had been diagnosed as diabetic were also excluded to ensure that there is no effect on the analysis arising from the strong relation between diabetes and obesity, or from the effect of a medical intervention that is not directly for obesity. Further exclusions were made for those with a BMI outside the range of 13–60 kgm^−2^, these units apply to all occurrences of BMI values throughout this paper. There were no exclusions made based on age. A total of 17009 adults were used for this study. The complete ENSANUT 2006 data has an approximate 2:1 ratio of women to men, within the filtered data in this analysis this ratio was reflected with 63.52% women and 36.48% men.

The condition of obesity was self-reported via the question: “Have you ever been told by a healthcare professional that you are obese?” From this point onwards, all references to identification refer to identified as obese by a healthcare professional. On the basis of the response to this question, four main groups based on gender and identification of obesity were constructed: Non-identified (NI) men [5933]; Identified (I) men [272]; Non-identified women [9954]; and Identified women [850]. In each group, all participants may, or may not, actually be obese.

**Fig. 1:**
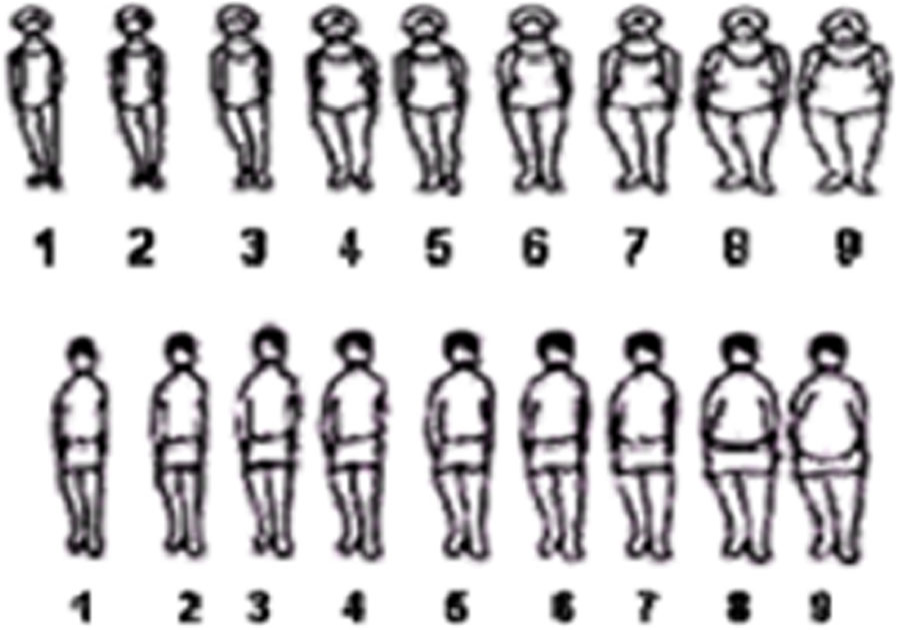
The figure rating scale in the form of a nine-figure Stunkard scale as used in the ENSANUT 2006 questionnaire to test the self-perception

Self-perception of BMI status was assessed using two distinct questions within the ENSANUT 2006 questionnaire [31]. The first was a categorical question, asking each individual to select one of four categories best describing their current weight. The options were: Underweight; Normal; Overweight; or Obese. The second question was image-based, using the Stunkard scale [32], as shown in Fig. 1 (ref. [31]). Alternative FRSs exist but the Stunkard 9-figure scale compares favourably to others [33]. This question asked each individual to select which figure best represents him or her at this moment.

**Fig. 2:**
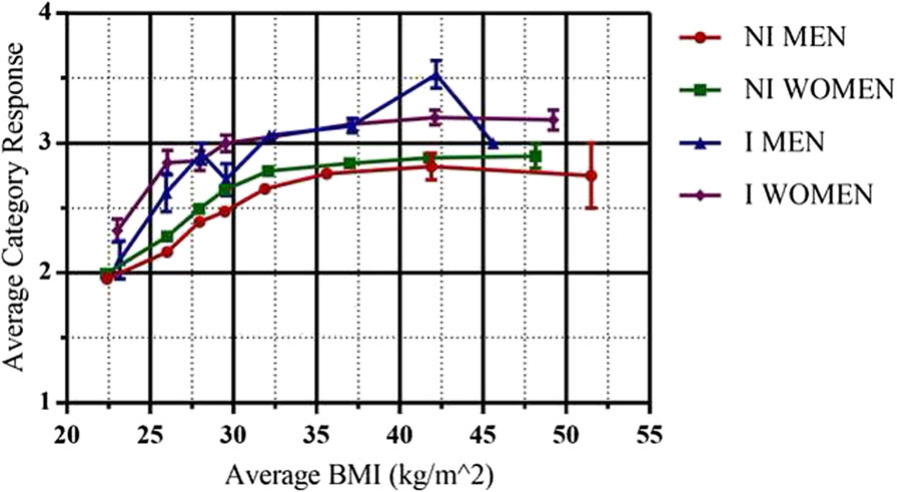
A comparison of mean perceived BMI (categorical) versus actual BMI, grouped by gender and identification of obesity by a medical professional. Average category response: 1 – underweight, 2 – normal, 3 – overweight, 4 – obese. NI – Non-identified, I – Identified. Standard error bars for average category response are included for each point

**Fig. 3:**
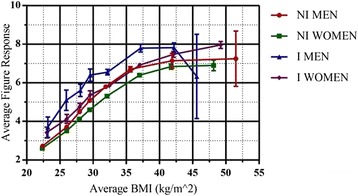
A comparison of mean figure rating scale response versus actual BMI, grouped by gender and identification of obesity by a medical professional. Average figure response from 1–9. NI – Non-identified, I – Identified. Standard error bars for average figure response are included for each point

For the purpose of comparison of averages for both questions, categories were converted to an ordinal scale as there is a clear size ordering. For the categorical question, we used the scale: underweight = 1, normal = 2, overweight = 3 and obese = 4. For the Stunkard scale, with figures 1–9, the standard categorisation is: 1–2 = underweight, 3–4 = normal, 5–7 = overweight and 8–9 = obese [34].

Actual weight was based on weight and height measurements obtained by trained health technicians to ensure measurement accuracy. BMI was calculated and standard weight categories were constructed. It is important to recognise that as both fat and muscle mass affect weight, it is possible to have a person with increased muscle mass and very little fat report a high BMI. We assume in this paper that for the general adult population BMI provides a good measure of weight and obesity.

In order to create fair comparisons across each of the four previously defined groups by gender and identification, one standardized set of coarse-grained bins was considered to group the data. Eight BMI bins were created with the following ranges: 13 ≤ BMI < 25, 25 ≤ BMI < 27, 27 ≤ BMI < 29, 29 ≤ BMI < 30, 30 ≤ BMI < 35, 35 ≤ BMI < 40, 40 ≤ BMI < 45 and 45 ≤ BMI ≤ 60. The binning was made to adhere to the boundaries of the main BMI cut-offs for underweight, normal, overweight and obese, in order to facilitate interpretation of the results. However, rather than only using the standard BMI categories eight BMI bins were created in order to have more discriminatory power as a function of BMI, while still maintaining a sufficiently large sample size in each bin. Thus, more/less bins means better/worse discrimination of the relation between actual and perceived BMI but worse/better statistical significance. Eight bins were chosen as a balance between these competing requirements. Due to the requirement of having the bins also respect the standard BMI categories it was not possible to guarantee equal sized bins for all, but the relative proportions ensure that each bin carries a substantial number for the analysis.

The average response to both BMI related questions were plotted with their standard error bars, for each of the four groups of gender and identification and for each of the eight BMI ranges. These graphs were used to visually assess the differences between identified and non-identified and genders.

To statistically verify the difference between two slopes, in this case for the non-identified and the identified, the interaction term regression method was used [35, 36]. By first calculating the slopes for the non-identified and the identified separately, we carried out a multiple regression incorporating two terms: an interaction term for the identified that is created by multiplying a categorical variable by the dependent variable; and self-perception response. These are compared against the actual BMI and it is the interaction term that is used to test the null hypothesis. Provided that the results of this test prove that there is no difference in the slopes, a comparison of the constants in the regression analysis can be made in order to test the statistical significance of the difference in the responses between the identified and the non-identified obese. This method involves using a categorical variable to distinguish between the identified and the non-identified obese in the regression. Should the result of the categorical variable be significant within the multiple regression, this would prove that there is a significant difference in the constants and hence indicate that the shift in self-perception is constant, due to the parallel nature of the slopes.

It is possible to determine that the difference in mean perceived BMI, as proxied by the metric scales, corresponds to a given difference in the means of actual BMI. To do this, the average responses on the metric scales, [1–4] for the categorical question and [1–9] for the FRS, were compared to give a measure of change in perception as BMI increases. Across the BMI ranges we are considering, the change in perception was compared to the change in actual BMI. We then determine what this change in actual BMI represents in the self-perception question scales. For example, by definition, if perceived BMI were a faithful measure of actual BMI, the categorical question should show an increase in average response from 2 to 4, as actual BMI increases from normal (18.5 ≤ BMI < 25, represented as 2) up to obese (30 ≤ BMI, represented as 4); an increase of two on the category scale. Similarly, for the FRS, an increase in actual BMI from normal to obese should correspond to an increase in figure response from 3–4 to 8–9. To convert the mean response into a measure of perceived BMI, we assume that the response scales are linear measures of BMI. By then carrying out a linear regression, the slope associated with perceived BMI was compared with the slope that best represents actual BMI change to determine the degree that differences in perception are an accurate estimate of real differences.

## Results

The frequency of obesity in non-identified men and women was 19.62 and 31.39% respectively, as shown in the summary statistics in Table 1. However, only 4.38 and 7.87% of men and women respectively had been identified by a healthcare professional as being obese. This corresponds to proportions of 18.94 and 21.38%, for men and women, who had been identified among those who are obese or had been previously identified as such. Of the identified, we can see in Table 1 that only 6.24% of women and 3.68% of men currently had a normal BMI (< 25), while 24.71 and 30.88%, respectively, changed from identified obese to actual overweight (25 ≤ BMI < 30).

**Table 1:** Summary statistics for the population of 17009 adults taken from ENSANUT 2006

NI Men			I Men			NI Women			I Women		
Normal	Over	Obese	Normal	Over	Obese	Normal	Over	Obese	Normal	Over	Obese
2219	2550	1164	10	84	178	3034	3795	3125	53	210	587
37.40%	42.98%	19.62%	3.68%	30.88%	65.44%	30.48%	38.13%	31.39%	6.24%	24.71%	69.06%

Primary categories are labelled by identification of obesity by a healthcare professional (*NI* – Non-Identified obese and *I* – Identified obese) and gender (Men, Women). Further division is based on actual BMI, where *Normal* represents 18.5 ≤ BMI < 25, *Over* represents 25 ≤ BMI < 30 and *Obese* represents BMI ≥ 30

As mentioned in the introduction, our hypothesis is that for both the men and women, there is a difference between the mean self-perception responses of a non-identified obese and an identified obese Mexican adult, for each BMI range, due to the effect of a consultation to identify obesity with a medical professional. Therefore, there are four main categories that were compared, the first two relate to the difference between non-identified and identified for the FRS for both men and women separately. The second two categories relate to the difference between the non-identified and the identified for the category self-perception question, again for men and women separately.

For each of the eight BMI ranges discussed in the methodology, and for both genders, the mean self-perception response was calculated for the non-identified and the identified. By taking the non-identified as the reference perspective, the comparison to identified adults would show the effect, if any exists, of identification. The results for the categorical opinion question can be seen in Fig. 2, with the results for the FRS in Fig. 3, where in both cases we have displayed the discrete, ordinal category and FRS variables as linear and metric.

In Fig. 2, we observed that the relationship between perceived BMI, as proxied by the category scale, and actual BMI is monotonic and non-linear, where the morbidly obese (BMI ≥ 35) did not show an increase in perceived BMI as a function of actual BMI irrespective of identification or gender.

We observed that the difference between responses for the identified and the non-identified is constant. This has been statistically confirmed by testing the null hypothesis that the slope for the non-identified is equal to the slope of the identified. The results for the analysis of the comparison of the two slopes for both, males (*p* = 0.773) and females (*p* = 0.614), showed that the differences in the slopes of the identified and non-identified are not statistically significantly different, hence, we fail to reject the null hypothesis. Thus, the relative misperception of BMI, defined as selecting a category/figure that does not correspond to actual BMI, is to a large degree independent of actual BMI. We also saw a systematic difference between the average category rating of non-identified men and women, with women reporting a slightly higher mean category rating than men.

Furthermore, the results shown in Fig. 2 clearly showed that a consultation to identify obesity systematically changes the self-perceived weight categorisation of the adults in the data set. This is inclusive of men and women and across all BMI ranges. Given that there was no significant difference between the slopes, indicating that the lines are parallel, the effect of a consultation was verified by a comparison of the constants from the regressions for the identified and the non-identified obese. The results for male responses to the category question were weakly significantly different (*p* = 0.052), and the results for the women were significantly different (*p* = 0.018). Full details on the regression analysis for the identified and non-identified men and women related to the category question are included in Table 2.

**Table 2:** Linear regression analyses of BMI versus category response for all gender/identification groupings for BMI < 35

	Variable	Unstd. B	Std. Error	t-statistic	f-statistic	R^2^	Sig	95% C.I. Lower	95% C.I. Upper
All					5764.888	0.271	0		
	Constant	19.384	0.101	192.18			0	19.186	19.581
	Category	3.224	0.042	75.927			0	3.141	3.307
Men					2173.229	0.269	0		
	Constant	19.01	0.162	117.302			0	18.692	19.328
	Category	3.303	0.071	46.618			0	3.164	3.442
Women					3473.562	0.266	0		
	Constant	19.68	0.13	151.671			0	19.426	19.934
	Category	3.15	0.053	58.937			0	3.045	3.255
NI					5826.474	0.268	0		
	Constant	18.255	0.125	146.304			0	18.01	18.499
	Category	3.996	0.052	76.331			0	3.893	4.099
Identified					93.45	0.109	0		
	Constant	24.302	0.591	41.146			0	23.143	25.462
	Category	1.923	0.199	9.667			0	1.532	2.313
Men NI					1916.112	0.251	0		
	Constant	19.119	0.167	114.695			0	18.792	19.445
	Category	3.229	0.074	43.773			0	3.085	3.374
Men I					29.848	0.127	0		
	Constant	24.214	1.083	22.351			0	22.078	26.25
	Category	2.006	0.367	5.463			0	1.282	2.73
Women NI					3092.896	0.255	0		
	Constant	19.685	0.134	146.771			0	19.422	19.948
	Category	3.121	0.056	55.614			0	3.011	3.231
Women I					64.919	0.103	0		
	Constant	24.31	0.702	34.624			0	22.931	25.689
	Category	1.9	0.236	8.057			0	1.437	2.363

In the Categories column the regression analyses are categorised by gender (All represents the combined group of men and women); and by identification of obesity (I and NI). Variable refers to the regression coefficients; *Unstd. B* Unstandardised Beta coefficient, *Std. Error* standard error, t-statistic, f-statistic, R^2^, *Sig* Significance value, *95% C.I. Lower* Lower bound of the 95% confidence interval, *95% C.I. Upper* Upper bound of the 95% confidence interval. Responses were taken only in the range BMI < 35 to test linearity

In Fig. 3 we observed that average figure rating is also a monotonically increasing, non-linear function of actual BMI for all groups. The only exception was the identified men in the range 45 ≤ BMI ≤ 60, where there are only three participants. As with the category scale, the morbidly obese (BMI ≥ 35) did not exhibit any increase in perceived BMI as actual BMI increased. Once again, this tendency was present for both identified and non-identified and both genders.

Again, the difference in the identified and the non-identified has been checked statistically via a comparison of the two slopes via an interaction term. For the FRS responses of both the males (*p* = 0.238), and the women (*p* = 0.815), the slopes of the non-identified and the identified were not statistically significantly different to each other, and thus the difference between identified and non-identified is constant. There was also a systematic difference by gender across both identification categories, with women being associated with a lower perceived BMI versus men for a given actual BMI.

We also saw that identification systematically changes the self-perception of both men and women on the FRS. The differences in self-perception responses for the FRS question between the identified and the non-identified have again been calculated using the comparison of constants in regression analysis. The comparison for identified and non-identified males showed that there was no significant difference across the full BMI range (*p* = 0.191), however, this is due to the sudden drop in the final BMI group for the diagnosed males. Carrying out this method, but excluding the final BMI range for males gives a highly significant result (*p* = 0.004). The results for the women across the full BMI range also gave a significant result (*p* = 0.017). There was an average increase across all BMI ranges that was approximately one figure for the men, while the effect on the women was slightly less, confirming that the relative misperception of BMI is to a large degree independent of actual BMI. Full details on the regression analysis for the identified and non-identified men and women related to the FRS question are included in Table 3.

**Table 3:** Linear regression analyses of BMI versus figure response for all gender/identification groupings for BMI < 35

	Variable	Unstd. B	Std. Error	t-statistic	f-statistic	R^2^	Sig	95% C.I. Lower	95% C.I. Upper
All					7573.014	0.328	0		
	Constant	22.306	0.057	388.213			0	22.193	22.148
	Figure	1.13	0.013	87.023			0	1.104	1.155
Men					2736.797	0.316	0		
	Constant	22.395	0.085	262.99			0	22.228	22.561
	Figure	0.982	0.019	52.314			0	0.945	1.019
Women					5126.825	0.348	0		
	Constant	22.132	0.076	290.36			0	21.983	22.281
	Figure	1.252	0.017	71.602			0	1.218	1.287
NI					6920.629	0.319	0		
	Constant	22.267	0.058	380.662			0	22.152	22.381
	Figure	1.12	0.013	83.19			0	1.094	1.147
Identified					168.078	0.18	0		
	Constant	26.031	0.318	81.938			0	25.407	26.655
	Figure	0.712	0.055	12.964			0	0.604	0.819
Men NI					2492.355	0.304	0		
	Constant	22.402	0.086	260.267			0	22.234	22.571
	Figure	0.964	0.019	49.923			0	0.927	1.002
Men I					31.447	0.132	0		
	Constant	26.551	0.65	40.817			0	25.269	27.834
	Figure	0.574	0.102	5.608			0	0.372	0.776
Women NI					4706.297	0.342	0		
	Constant	22.053	0.078	282.442			0	21.9	22.206
	Figure	1.254	0.018	68.602			0	1.218	1.29
Women I					140.332	0.201	0		
	Constant	25.759	0.369	69.77			0	25.033	26.484
	Figure	0.786	0.066	11.846			0	0.656	0.917

In the Figures column the regression analyses are categorised by gender (All represents the combined group of men and women); and by identification of obesity (I and NI). Variable refers to the regression coefficients; *Unstd. B* Unstandardised Beta coefficient, *Std. Error* standard error, t-statistic, f-statistic, R^2^, *Sig* Significance value, *95% C.I. Lower* Lower bound of the 95% confidence interval, *95% C.I. Upper* Upper bound of the 95% confidence interval. Responses were taken only in the range BMI < 35 to test linearity

From Figs. 2 and 3 we observed visually that there was a substantial range of BMI, BMI < 38 for the FRS and BMI < 32 for the category question, over which the relationship between BMI and the corresponding integer-mapped metric scales for the category question and FRS were approximately linear. We investigated further the linearity of the curves, over the interval BMI < 35, by performing linear regressions – FRS/category response versus BMI–of the raw data for the following categories: All adults, all men, all women, all identified, all non-identified, identified men, non-identified men, identified women and non-identified women. As necessary when using linear regression, the assumptions of a linear relationship between the two variables, a lack of outliers, independence of observations, homoscedacity and the residuals of the regression line being normally distributed were all checked and verified. SPSS Statistics 22 was used for all aspects of statistical analysis. The results can be seen in Table 2 for the category and in Table 3 for the FRS question.

Notably, for both figures and categories, we observed that all regressions were statistically significant, with f-statistics in the range 31.80–7573.01 and t-statistics for the regression coefficients in the range 5.64–87.02, with lower values being associated with the identified, where the sample sizes were smaller.

## Discussion

The relationship between perceived BMI and actual BMI exhibits some universal features across the two distinct self-perception questions. First of all, the monotonicity of the relations showed that, as would be hoped, on average, those who had greater BMI also perceived themselves to have greater BMI. Furthermore, over a substantial range of actual BMI the relation was approximately linear for both the FRS and the category scale. The question then becomes: Is the rate of increase of perceived BMI equal to the rate of increase of actual BMI? What would one expect as an appropriate rate of increase of perceived BMI as a function of actual BMI if there were no weight misperception? As stated, the standard categorisation of the Stunkard scale figures are as follows; 1–2 = underweight, 3–4 = normal, 5–7 = overweight and 8–9 = obese [34]. If we take the FRS to be a linear measure of BMI, one would expect BMI to increase from approximately 20 to approximately 30 as the FRS ranges from 3 to 8, i.e., 2 kgm^−2^ per unit increase in figure rating. If we conservatively consider only four figures to cover this BMI range, then one would determine 2.5 kgm^−2^ per unit increase in figure rating. Similarly, if we consider a more extensive range of figures, say 6, then one would expect a rate of change of 1.67 kgm^−2^ per unit increase in figure rating. For the categorisation question, if the respondents perfectly classified their BMI status, then the normal group (18.5 ≤ BMI < 25) should have an average category rating of 2, the overweight (25 ≤ BMI < 30) an average of three and the obese (BMI ≥ 30) an average of 4, i.e., a monotonic increase from 2 to 4 over the range 18.5 ≤ BMI < 25, corresponding to a slope of 5.75.

In Fig. 3, and quantitatively in Table 3, we observed that the range of slopes across all groups is in the range 0.574–1.254. These values are far below what would be expected if BMI perceptions were faithfully following actual BMI. As the maximum slope corresponding to the 95% confidence intervals was 1.29, we can state that no slope is consistent with any of the null hypotheses of 1.67, 2 or 2.5 kgm^−2^ per unit change in figure rating. Thus, we saw that the relative misperception of BMI, understood as the difference between the change in perceived BMI per unit increment in actual BMI, was fairly constant over a substantial range. Indeed, in the case of the FRS, the linearity extended to the onset of morbid obesity (BMI ≥ 35). Beyond this point, the increase in perceived BMI per unit increase in actual BMI decreased even further, such that there was only a very small increase in perceived BMI – approximately one figure rating unit – over a very wide range, 35 ≤ BMI < 50.

For the categorical question, the expected slope is 5.75 while, as we can see in Table 2, the observed slopes were in the range 1.9 to 4, i.e., between 67 and 30% less. Once again, there was a qualitative change in behaviour around the onset of morbid obesity. In the context of the categorical question, this is an indication that the proportion of morbidly obese respondents, who consider themselves to be obese, is small. In fact, in the BMI ranges 35 ≤ BMI < 40, 40 ≤ BMI < 45 and 45 ≤ BMI ≤ 60, the fractions that considered themselves to be obese were only 6.69, 11.04 and 13.95% respectively.

The large discrepancy between the slope of the expected relation between perceived and actual BMI and the actual relation is a clear indication that there is a large misperception between perceived and actual BMI differences. A given BMI increment was perceived as between 25 and 77% less than its real value, the precise value depending on how a figure rating increment, or category increment, is translated into a perceived BMI increment and which subgroup we consider. The principal implication is that any given BMI increase is perceived as considerably less than it really is. Misperception of BMI has standardly been emphasized with respect to the misperception of the obese and overweight. What we have demonstrated here is that our results are consistent with a relative misperception of BMI that is both large and independent of BMI over a substantial BMI range.

Why could relative misperception of BMI be constant? We believe that the universal form of the relationship between perceived and actual BMI, is an important indicator of the underlying psychology of weight perception and hypothesise that it is linked to the self-serving bias [37], a well-established cognitive bias strongly linked to self-esteem. This self-serving bias acts, we argue, to consistently underestimate BMI increments independently of actual BMI.

A further notable feature of Figs. 2 and 3, seen in detail in Tables 2 and 3, is the difference in perception between the non-identified and the identified and between genders. Within our linear approximation, such differences in perception manifest in differences in the regression coefficients – either the slope or the constant. From Table 3, we saw that the difference in constants, 22.27 versus 26.31, was statistically significant between the identified and non-identified, as is the difference between the slopes, 1.12 versus 0.712. These conclusions are also valid when comparing both identified and non-identified men and women as a sub-category. For the categorical question, we also saw that there is a noticeable difference between the identified and non-identified by gender. Interestingly, there was no statistically significant difference in either intercept or slope between identified women and identified men. Similarly, there was no statistically significant difference between the intercepts for non-identified men and women but there was between slopes. It is worth noting that across every pair of comparable categories the intercept of the identified was greater than that of the non-identified and the slope less. Thus, we can see that identification of obesity is consistent with a shift in perception that manifests itself principally as a constant shift in perceived BMI. This was seen most clearly for BMI < 25, where it was associated with a difference of 3–4 kgm^−2^ for the same figure rating. In this regime, it is clearly identifiable as an over-assessment of BMI of the identified relative to the non-identified. However, as the regression coefficients were less for an identified group than the corresponding non-identified group it implies that the identified perceived an actual BMI change as being less than that perceived by the non-identified.

We propose that this change can be understood in terms of another cognitive bias – anchoring [38] – that occurs when individuals use an “initial” piece of information in making subsequent judgments. If an anchor has been set, then judgments are biased in that they adjust away from that anchor. We propose that identification acts as an anchor, from which subsequent self-perception of BMI is measured. Thus, anchoring is a natural explanation of the offset between the identified and the non-identified curves in the figures and is additional to the self-serving bias, which we claim is an important element in the shape of the curves. It is also consistent with the smaller perceived effect of a BMI change for the identified if we take the anchoring effect as to anchor the identified to the obese category, therefore perceiving weight loss in terms of figure ratings to be less than it actually is.

It has been argued that identification leads to a more accurate self-perception of BMI, in the case of the obese [17, 29]. However, it has also been shown that over-assessment of weight is prevalent [13]. Above, we argued that an identification of obesity has the effect of increasing the degree of over-assessment of weight given that only 8.91% of the non-identified with BMI < 25 considered themselves to be overweight or obese, with the corresponding figure for the identified being 36.51%. This is consistent with what is observed in Figs. 2 and 3. Thus, we assert that identification does not lead to a superior capacity to assess BMI per se but, rather, simply leads to a different anchoring point from which BMI is assessed. The apparent improvements in perception of BMI associated with higher fractions of the obese recognising their obese status after an identification we would argue relates to the anchoring effects of the identification coupled with the fact that so few obese return to a normal weight. A more accurate self-perception of body weight and BMI and, importantly, changes in bodyweight, would be very advantageous in the fight against obesity. Here the belief is that while it is not guaranteed to result in action, knowledge and acceptance of a problem is the crucial first step to making a change.

The final difference we note is that between genders. This was seen in Tables 2 and 3, where we noticed that the slopes for women were statistically significantly greater than those for men in the case of the non-identified. Thus, non-identified women/men over-assess/under-assess their weight relative to men/women. This relative over-assessment of weight reduces as a function of BMI, exhibiting the fact that non-identified women perceived a given BMI increment as being bigger than that perceived by a non-identified man. For the category question there were no statistically significant differences between identified/non-identified men and women.

Some potential limitations of this research are: i) the data is cross-sectional, thereby making causal inference more difficult; this is particularly the case in that we are considering BMI differences across different group not across a cohort of the same individuals; ii) the male/female ratio for the ENSANUT respondents is skewed and therefore not fully representative of the Mexican population; iii) identification of obesity by a healthcare professional was self-reported and therefore subject to recall and other biases; iv) Weight categories are defined using standard BMI cut-off points and these are potentially unknown to most respondents; v) We assume that no other variable distinguishes the identified from the non-identified, e.g. socio-economic category etc. However, we checked explicitly that the age distribution was the same; vi) We have assumed that the Stunkard scale and the category scale can be interpreted as approximately linear measures of BMI.

## Conclusions

An impressive aspect of the obesity epidemic is its universality–crossing gender, racial, genetic and cultural boundaries, to name but a few – the differences between these groups being notably smaller than the overall effect. Although obesity is a highly multi-factorial problem, this hints at the fact that universal tendencies exist, which are to a large degree independent of these factors. We suggest that various cognitive biases could be one source of such tendencies and therefore could play a potentially important role in the obesity epidemic. Here, we have mentioned two: the self-serving bias and anchoring bias; that we argue could be responsible for our results. Recent results have also commented on these cognitive biases for self-perception but in relation to the ENSANUT 2012 database [39].

We believe that the results we have shown have important ramifications for public health interventions. It is accepted that an important step for implementing a weight loss program is for the person to accept their obese status and, preferably, to accept it as a serious condition. In this case, emphasis is squarely placed on the concept of weight/BMI status and its recognition. Misperception of BMI is then a severe obstacle to this goal. There is, however, no clear evidence that such identifications lead to long-term reductions in BMI through weight reduction when compared to the non-identified. The difficulty of returning to a normal weight from obesity have recently been emphasised by Fildes et al. [40], where over a 9-year period 3.1% of obese (30 ≤ BMI < 35) men and 5.1% of obese women returned to a normal BMI. These numbers are consistent with our results, 3.68 and 6.24% respectively, in that they show the immense challenges associated with returning to a healthy BMI.

We believe that the public health message of this paper is, rather, that the most important impact of misperceptions of BMI is not in gauging BMI state but in terms of perceiving and gauging changes, both positive and negative, in BMI. Uniformly, BMI changes are underestimated, with the identified being particularly susceptible. For instance, an actual BMI increment of 2 kgm^−2^ may be perceived to be an increment of just half that value. Thus, the real danger is in underestimating weight increases until it is too late. This is consistent with a preventative approach, implementing wide-ranging screening to detect those whose BMI has increased substantially.

## Abbreviations

BMI: Body mass index
ENSANUT: National Survey of Health and Nutrition [from “Encuesta Nacional de Salud y Nutrición” in Spanish]
FRS: Figure rating scale
I: Identified
NI: Non-identified
